# Enhancing Hi-C contact matrices for loop detection with Capricorn: a multiview diffusion model

**DOI:** 10.1093/bioinformatics/btae211

**Published:** 2024-06-28

**Authors:** Tangqi Fang, Yifeng Liu, Addie Woicik, Minsi Lu, Anupama Jha, Xiao Wang, Gang Li, Borislav Hristov, Zixuan Liu, Hanwen Xu, William S Noble, Sheng Wang

**Affiliations:** Paul G. Allen School of Computer Science and Engineering, University of Washington, Seattle, WA 98195, United States; Paul G. Allen School of Computer Science and Engineering, University of Washington, Seattle, WA 98195, United States; Paul G. Allen School of Computer Science and Engineering, University of Washington, Seattle, WA 98195, United States; Paul G. Allen School of Computer Science and Engineering, University of Washington, Seattle, WA 98195, United States; Department of Genome Sciences, University of Washington, Seattle, WA 98195, United States; Department of Computer Science, Purdue University, West Lafayette, IN 47907, United States; Department of Genome Sciences, University of Washington, Seattle, WA 98195, United States; eScience Institute, University of Washington, Seattle, WA 98195, United States; Department of Genome Sciences, University of Washington, Seattle, WA 98195, United States; Paul G. Allen School of Computer Science and Engineering, University of Washington, Seattle, WA 98195, United States; Paul G. Allen School of Computer Science and Engineering, University of Washington, Seattle, WA 98195, United States; Paul G. Allen School of Computer Science and Engineering, University of Washington, Seattle, WA 98195, United States; Department of Genome Sciences, University of Washington, Seattle, WA 98195, United States; Paul G. Allen School of Computer Science and Engineering, University of Washington, Seattle, WA 98195, United States

## Abstract

**Motivation:**

High-resolution Hi-C contact matrices reveal the detailed three-dimensional architecture of the genome, but high-coverage experimental Hi-C data are expensive to generate. Simultaneously, chromatin structure analyses struggle with extremely sparse contact matrices. To address this problem, computational methods to enhance low-coverage contact matrices have been developed, but existing methods are largely based on resolution enhancement methods for natural images and hence often employ models that do not distinguish between biologically meaningful contacts, such as loops and other stochastic contacts.

**Results:**

We present Capricorn, a machine learning model for Hi-C resolution enhancement that incorporates small-scale chromatin features as additional views of the input Hi-C contact matrix and leverages a diffusion probability model backbone to generate a high-coverage matrix. We show that Capricorn outperforms the state of the art in a cross-cell-line setting, improving on existing methods by 17% in mean squared error and 26% in *F*1 score for chromatin loop identification from the generated high-coverage data. We also demonstrate that Capricorn performs well in the cross-chromosome setting and cross-chromosome, cross-cell-line setting, improving the downstream loop *F*1 score by 14% relative to existing methods. We further show that our multiview idea can also be used to improve several existing methods, HiCARN and HiCNN, indicating the wide applicability of this approach. Finally, we use DNA sequence to validate discovered loops and find that the fraction of CTCF-supported loops from Capricorn is similar to those identified from the high-coverage data. Capricorn is a powerful Hi-C resolution enhancement method that enables scientists to find chromatin features that cannot be identified in the low-coverage contact matrix.

**Availability and implementation:**

Implementation of Capricorn and source code for reproducing all figures in this paper are available at https://github.com/CHNFTQ/Capricorn.

## 1 Introduction

Chromosomes encode genetic and epigenetic cellular programs, leveraging a complex three-dimensional (3D) genome architecture in eukaryotic cells that is critical for many biological processes, including modulating gene regulatory relationships, RNA splicing sites, and DNA repair mechanisms (Cremer and Cremer 2001, Bonev and Cavalli 2016). These architectures within a cell can be measured with assays such as high-throughput chromosome conformation capture (Hi-C) (Lieberman-Aiden *et al.* 2009), genome architecture mapping (GAM) (Beagrie *et al.* 2017), split-pool recognition of interactions by tag extension (SPRITE) (Quinodoz *et al.* 2018), and HiChIP (Mumbach *et al.* 2016). *High-resolution* Hi-C datasets employ smaller bin sizes, thereby more precisely characterizing genomic substructures, but consequently require more experimental reads to produce a suitably dense matrix. Importantly, doubling the resolution requires quadrupling the experimental read counts due to the pairwise nature of interactions (Schmitt *et al.* 2016).

To obtain denser contact matrices, computational *resolution enhancement* methods take low-coverage, high-resolution Hi-C matrices with fewer measured contacts and generate the corresponding high-coverage, high-resolution matrices. Existing approaches adopt techniques from computer vision, such as convolutional neural networks (CNNs) (Zhang *et al.* 2018, Liu and Wang 2019a, 2019b, Li and Dai 2020), generative adversarial networks (GANs) (Liu *et al.* 2019, Dimmick 2020, Hong *et al.* 2020, Highsmith and Cheng 2021, Hicks and Oluwadare 2022), and Markov random fields (MRFs) (Cameron *et al.* 2020) to produce detailed contact matrices. The resulting data can then be used to analyze genome folding, including classification of large-scale chromatin features like A/B compartments (Lieberman-Aiden *et al.* 2009, Chakraborty *et al.* 2022) and identification of small-scale chromatin features like topologically associating domains (TADs) (Dixon *et al.* 2012, Filippova *et al.* 2014, Crane *et al.* 2015) and chromatin loops (Rao *et al.* 2014, Roayaei Ardakany *et al.* 2020). Although existing approaches aim to minimize mean-squared error (MSE) and perform well according to metrics developed for natural image analyses, these metrics may not be well suited to teach the model to capture biologically relevant chromatin features, and especially small-scale loops.

**Figure 1.**
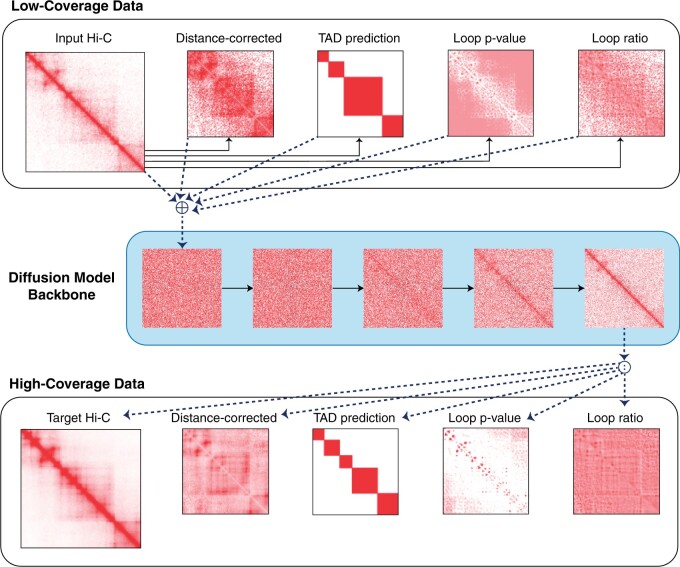
Overview of Capricorn architecture. Given the low- and high-coverage Hi-C contact matrices we compute small-scale chromatin features that explicitly teach Capricorn to recognize biologically meaningful contacts. We then use a diffusion model backbone to iteratively de-noise a random contact matrix, conditioned on the low-coverage data

We hypothesize that resolution enhancement can produce contact matrices that better capture these higher-order chromatin structures if we design a loss function that explicitly models structures like loops and TADs during resolution enhancement (Table 1). Such an approach can both provide additional supervision for the resolution enhancement task and help teach the model to distinguish and enhance particularly interesting genomic contacts. We propose Capricorn, which incorporates additional biological views of the contact matrix to emphasize important chromatin interactions and leverages powerful diffusion models from computer vision (Saharia *et al.* 2022) for the model backbone (Fig. 1). In particular, Capricorn learns a diffusion model that enhances a five-channel image, containing the primary Hi-C matrix as well as representations of loops, TADs, and distance-normalized counts computed from the low-coverage matrix. Capricorn thereby learns meaningful structural contacts as well as the overall matrix structure.

**Table 1.** A summary of existing Hi-C resolution enhancement methods.

Method	Publication date	Pixel-wise loss	Adversarial loss	TV loss	Perceptual loss	Biology	Backbone
HiCPlus	Feb. 2018	*✓*					CNN
hicGAN	July 2019		*✓*				CNN (GAN)
HiCNN2	Oct. 2019	*✓*					CNN
HiCNN	Nov. 2019	*✓*					CNN
HIFI	Jan. 2020					*✓*	MRF
DeepHiC	Feb. 2020	*✓*	*✓*	*✓*	*✓*		CNN (GAN)
SRHiC	Apr. 2020	*✓*					CNN
HiCSR	July 2020	*✓*	*✓*		*✓*		CNN (GAN)
VEHiCLE	Apr. 2021	*✓*	*✓*			*✓*	CNN (GAN)
EnHiC	July 2021	*✓*	*✓*		*✓*		CNN (GAN)
HiCARN-1	Apr. 2022	*✓*		*✓*	*✓*		CNN
HiCARN-2	Apr. 2022	*✓*	*✓*	*✓*	*✓*		CNN (GAN)
**Capricorn**	(ours)	*✓*				*✓*	Diffusion

We indicate whether each method adopts common computer vision loss terms, with “TV” for total variation and where pixel-wise losses include mean squared error (MSE) and L1 loss. Notably, only VEHiCLE (Highsmith and Cheng 2021), HIFI (Cameron *et al.* 2020), and Capricorn incorporate biological features into the resolution enhancement model. Although VEHiCLE uses TAD identification in its loss and HIFI accounts for TAD boundaries during local smoothing, Capricorn is trained to enhance loop and TAD features along with the contact matrix. Capricorn is the only method that uses a diffusion model as the backbone. We limit the comparison to methods for Hi-C resolution enhancement for proximal contacts (interaction within 2 megabases) that do not require additional high-coverage input data, excluding distal-contact enhancement techniques like BoostHiC (Carron *et al.* 2019) and reference-based methods like RefHiC-SR (Zhang and Blanchette 2023).

We compare Capricorn to four existing Hi-C resolution enhancement approaches representing the state of the art, and we find that Capricorn outperforms the others in terms of both MSE and its ability to detect loops from the predicted high-coverage contact matrix. We tested the models’ generalizability across both cell line and chromosome and found that Capricorn’s enhanced matrices had a 17.3% lower MSE and 25.6% higher loop *F*1-score on average when transferring learned patterns across cell lines. We further found that Capricorn’s key idea of incorporating higher-order chromatin features as additional input views is a broadly applicable technique that improved the comparison approaches as well, though Capricorn’s diffusion model backbone still provides the best performance. As a final validation, we use DNA sequence to evaluate the fraction of identified loops from the high coverage, low coverage, and Capricorn-generated contact matrices that are supported by flanking CTCF motifs, and find that Capricorn’s loops have comparable CTCF support to the high-coverage-derived loops. In summary, Capricorn is a general-purpose approach for chromatin conformation capture contact matrix resolution enhancement, and in the future additional feature views can be easily incorporated into the framework for various downstream tasks of interest.

## 2 Materials and methods

### 2.1 Problem setting

In the supervised resolution enhancement task, we are given a dataset with *N* pairs of low- and high-coverage contact matrices ${\left\{(X_{i},Y_{i})\right\}}_{i=1}^{N}$. For a fixed resolution Δ defining the size of each grouped genomic locus and a chromosome length of *L* base pairs, the contact matrix shapes are identical, with $X,Y\in N^{L/\Delta \times L/\Delta }$. However, the high-coverage matrix **Y** contains *γ*-fold more measured contacts in the matrix. The aim is to obtain a model $f_{\theta }$ such that $Y_{i}\approx f_{\theta }(X_{i})$. Furthermore, we want $f_{\theta }(\cdot )$ to generalize to both new cell lines and new chromosomes.

We process the input Hi-C matrices for efficient and structurally informative resolution enhancement. Most Hi-C interactions occur between nearby intrachromosomal loci. (Rao *et al.* 2014) As we focus on small-scale chromatin loops, we follow previous work in resolution enhancement (Liu *et al.* 2019, Hicks and Oluwadare 2022) and restrict the model to enhance intrachromosomal contacts within 2 Mb. For computational efficiency, we further tile paired contact matrices’ near-diagonals into 40  ×  40 non-overlapping matrices using a resolution Δ = 10 kb such that each input covers a 400^2^ kb region of contacts, consistent with many existing Hi-C resolution frameworks (Zhang *et al.* 2018, Liu and Wang 2019a, 2019b, Liu *et al.* 2019, Dimmick 2020, Hong *et al.* 2020, Li and Dai 2020, Hicks and Oluwadare 2022).

### 2.2 Chromatin structure resolution enhancement

The key idea behind Capricorn is that explicitly modeling small-scale chromatin features such as loops and TADs will improve the method’s ability to identify and enhance meaningful contacts. We therefore train Capricorn to enhance the structural interpretation of the low-coverage contact matrix as well as the low-coverage contact matrix itself, thereby explicitly training the model to recognize important 3D chromatin structures from low-coverage data.

Toward this end, we compute additional views of the paired (X,Y) contact matrices using chromatin features derived from **X** and **Y**. Additional details are provided in Supplementary Section S2.

1. Distance-corrected: $\left(X^{\left(oe\right)},Y^{\left(oe\right)}\right)$. Hi-C contact matrices contain a contact bias based on inter-loci distance, resulting in patterns like the strong signal along the matrix diagonal (Fig. 1). For this view, we divide the experimental contact matrix by an expected contact map based on distance, thereby identifying contacts that are more or less strongly measured than would be expected given inter-loci distance.
2. Called loops: $\left(X^{\left(\mathrm{loop}-p\right)},X^{\left(\mathrm{loop}-r\right)},Y^{\left(\mathrm{loop}-p\right)},Y^{\left(\mathrm{loop}-r\right)}\right)$. Given a contact matrix, we use the Hi-C Computational Unbiased Peak Search (HiCCUPS) algorithm (Rao *et al.* 2014) to identify chromatin loops and explicitly incorporate chromatin structure into the enhancement problem. The HiCCUPS algorithm considers whether measured contacts in the distance-corrected experimental matrix are significantly more frequent than the surrounding neighborhood, computing both a loop ratio of measured contacts relative to the experimental background and a *loop P-*value indicating the significance of this enrichment. We incorporate HiCCUPS’s loop ratio and *P*-value as two additional views for each input contact matrix.
3. TAD score: $(X^{\left(\mathrm{tad}\right)},Y^{\left(\mathrm{tad}\right)}$. We use the insulation score (IS) (Crane *et al.* 2015) to identify TADs in the input contact matrices. As the average human TAD is approximately 1 Mb long (Rao *et al.* 2014, Dali and Blanchette 2017), this view provides chromatin context that extends beyond a single tiled 400^2^ kb^2^ submatrix, thereby providing the resolution enhancement model information about the greater surrounding chromatin structure.

Importantly, we compute each of the low-coverage chromatin features directly from the low-coverage experimental matrix **X**, so there is no leakage between the high-coverage contact matrix and low-coverage inputs to Capricorn, which would prevent Capricorn’s practical utility during inference. The original contact matrices and derived chromatin structures are then concatenated to form the full input and output

(1) $$\begin{array}{c} \tilde{X}(X)=[X,X^{\left(oe\right)},X^{\left(\mathrm{tad}\right)},X^{\left(\mathrm{loop}-p\right)},X^{\left(\mathrm{loop}-r\right)}]\\ \tilde{Y}(Y)=[Y,Y^{\left(oe\right)},Y^{\left(\mathrm{tad}\right)},Y^{\left(\mathrm{loop}-p\right)},Y^{\left(\mathrm{loop}-r\right)}], \end{array}$$

such that $\tilde{X}(X),\tilde{Y}(Y)\in R^{5\times L/\Delta \times L/\Delta }$.

### 2.3 Multiview weighting

Each of the computed biological views naturally has a different distribution of values and presents different challenges for correct prediction. To correct for these differences, we perform a two-stage iterative view-weighting process based on the low-coverage representation $\tilde{X}(X)$ (Supplementary Section S3). To compute initial weights $\omega_{0}\in R{+}^{5}$, we normalized the distribution of each view by dividing the standard deviation of the original Hi-C experimental view **X** by the standard deviation of all other channels. We then further refined the weight vector by conducting an initial end-to-end run of Capricorn where each input and output view was multiplied by the corresponding term in $\sqrt{\omega_{0}}$. We computed the final weights $\omega \in R{+}^{5}$ by normalizing for the difficulty of generating each view, using the MSE loss computed over each view in the validation set to approximate difficulty. After these two initial runs to tune *ω*, we subsequently trained the full version of Capricorn by weighting each view by $\sqrt{\omega }$ when computing both the inputs and outputs.

### 2.4 Review of diffusion models

We use a *diffusion model* backbone (Sohl-Dickstein *et al.* 2015, Ho *et al.* 2020) to carry out the resolution enhancement task given the low-coverage contact matrix and derived chromatin feature views. Diffusion models are easier to train than the generative adversarial networks and recently have been shown to excel in image generation tasks (Li *et al.* 2022, Gao *et al.* 2023, Saharia *et al.* 2023). To the best of our knowledge, Capricorn is the first approach for Hi-C resolution enhancement that leverages diffusion models, and we therefore provide a brief overview here with more details in Supplementary Section S4.

Diffusion models leverage a latent Markov chain framework that iteratively denoises an input to produce diverse and realistic outputs (Sohl-Dickstein *et al.* 2015, Ho *et al.* 2020). The models are split into a *T*-step *forward process* $q(y_{t}|y_{t-1})$ and *T*-step *reverse process* $p(y_{t-1}|y_{t},x)$, where $y_{0}\in R^{C\times W\times H}$ is the *C*-channel original, high-fidelity image, $y_{t}\in R^{C\times W\times H}$ is a latent variable, and **x** is an optional input term on which to condition the process.

We specifically focus on *conditional* diffusion models trained with a MSE objective in the pixel space of an image, or the bin space of a contact matrix. We compute the expected MSE loss between the paired target image and generated image over each step in the forward- and reverse-processes, including the original input image $y_{t}$ and the final generated output ${\hat{y}}_{t}$.

### 2.5 Low-coverage guided diffusion

We leverage conditional diffusion probability models for the resolution enhancement task. The combined contact matrix and its derived chromatin feature views can be interpreted as a five-channel image, so we approach the problem as high-coverage image generation where low-coverage inputs used to guide the generation process. Specifically, Capricorn is built around a module that approximates a denoising step as part of the reverse process (Fig. 1).

Capricorn’s multichannel framework encodes additional biological understanding of the contact matrix data into the diffusion model resolution enhancement task, teaching the model to identify significant contacts while enabling the standard MSE loss formulation. Specifically, compute the MSE loss over all five biological views of the output, therefore treating $\tilde{X}(X)$ and $\tilde{Y}(Y)$ as five-channel source and target images, respectively. Furthermore, during inference we are able to use the trained diffusion model backbone and low-coverage contact matrix views to generate a high-coverage contact matrix estimate $\hat{\tilde{Y}}∼f_{\theta }(\theta ,\tilde{X}(X))$. After inference, we discard all views except for the enhanced Hi-C contact matrix (the 0th image channel) to produce $\hat{Y}\in R^{L/\Delta \times L/\Delta }$.

### 2.6 Performance measures

We evaluate the results using both image-based and biologically motivated metrics, focusing on the generated high-coverage contact matrix. Specifically, given a model’s predicted contact matrix $\hat{Y}$, we compute the MSE as ${\left\|\hat{Y}-Y\right\|}_{2}^{2}$.

We also measure the structural accuracy of the generated high-coverage contact matrix, focusing on chromatin loops. We use the enhanced high-coverage matrix to call loops with the HiCCUPS algorithm (Rao *et al.* 2014), which uses four different kernels to compare measured contacts with the contacts in the local neighborhood and test for enrichment, producing both an enrichment ratio and *P*-value. We annotate all loci (*i*, *j*) with *P*-value < 0.1 and enrichment ratio > 1.75 for HiCCUPS’s donut or lower-left-quadrant kernels or > 1.5 for HiCCUPS’s horizontal or vertical kernels as a loop, following the default tool parameters (see Supplementary Section S2.2 for more details). As HiCCUPS is the algorithm that we also use for loop-based view computation, we additionally call loops with the Mustache (Roayaei Ardakany *et al.* 2020) and Chromosight (Matthey-Doret *et al.* 2020) loop-detection tools during evaluation. Mustache identifies loops using computer vision approach that considers loop features at multiple resolutions, and Chromosight leverages a loop template to detect similar patterns in a given contact matrix. For all tools, we use the default parameters for loop detection.

For each loop calling tool, we compute a loop *F*1 score $F1=\frac{TP}{TP+\frac{1}{2}(FP+FN)}$ with a 5 pixel tolerance range, such that *TP* are the true positive loops called from the predicted data that appear within [*i*–5: *i *+* *5, *j*–5: *j *+* *5] in the loops called from the ground-truth data, *FP* are the loops called from the predicted data that do not appear within the five-pixel range from the ground-truth data, and *FN* the loops that are called from the ground-truth data but do not occur in the five-pixel tolerance range for predicted data. We do not use true negatives in our evaluations, as this would include most of the genome and not be an informative metric.

### 2.7 Hi-C data preprocessing

We collected Hi-C data for the GM12878 Epstein–Barr–virus-infected human lymphoblastoid cell line and the K562 human chronic myelogenous leukemia lymphoblast cell lines from Rao *et al.* (2014) (Supplementary Section S1), restricting the contact matrix to read mapping quality ≥30 and processed to 10 kilobase (kb) resolution following previous work (Hicks and Oluwadare 2022). We adopt the contact matrix preprocessing techniques from HiCARN (Hicks and Oluwadare 2022) and DeepHiC (Hong *et al.* 2020), including clamping the high-coverage matrix to [0,255], and then, normalizing to [0,1], and clamping the low-coverage matrix to [0,100] and then normalizing to [0,1].

To simulate low-coverage data, we randomly downsampled the GM12878 and K562 cell line Hi-C matrices (Rao *et al.* 2014) to $\frac{1}{16}$ of their original read count. We treated these downsampled data as the low-coverage matrices and the original matrices as their high-coverage pairs. We evaluate model performance in a cross-cell-line setting where we train on either the GM12878 data or K562 data and test the model on the other cell line. In both experiments, we withhold chromosomes 4, 5, 11, and 14 from the training cell line as our validation set. Chromosome 9 is also excluded from the K562 data due to extreme sparsity at 10 kb resolution.

### 2.8 Existing model implementations

We use the publicly available python HiCARN (Hicks and Oluwadare 2022) repository from https://github.com/OluwadareLab/HiCARN for both their model and contact matrix preprocessing implementation, as well as source code implementations for HiCNN (Liu and Wang 2019a) and HiCSR (Dimmick 2020). We retrained all comparison models on our data. We reimplemented HiCCUPS (Rao *et al.* 2014) to accept NPZ input files, enabling loop calling for low-coverage and generated contact matrices that had a fixed resolution. We verified that our implementation was reasonable by comparing the number of called loops on the high-coverage, primary GM12878 Hi-C matrix used in our experiments (Rao *et al.* 2014). In total, we call 10 179 loops compared to the default implementation identifying 7949 loops. We also find comparable CTCF support (Handoko *et al.* 2011) for our called chromatin loops as the original data reported in Rao *et al.* (2014) (see Section 3, Table 2). We used the available Python implementation of Mustache at https://github.com/ay-lab/mustache (Roayaei Ardakany *et al.* 2020) and the packaged version of Chromosight at https://pypi.org/project/chromosight/ (Matthey-Doret *et al.* 2020).

**Table 2.** Analysis of CTCF-validated loops for high-coverage, low-coverage, and Capricorn-generated contact matrices.

Cell line	Contact matrix	Total loops	Validation rate (%)
GM12878	High-coverage	10 176	41.1
	Low-coverage	7029	24.7
	Capricorn	5798	42.8
K562	High-coverage	5142	37.6
	Low-coverage	4668	15.8
	Capricorn	2223	36.8

We show the total number of called loops and the percentage of called loops that also have DNA CTCF support.

We also use the conditional diffusion probability model Imagen (Saharia *et al.* 2022) as the resolution enhancement backbone model, updating the model to condition on low-coverage contact matrices rather than text. We choose Imagen rather than other image diffusion models (Nichol *et al.* 2021, Rombach *et al.* 2021, Ramesh *et al.* 2022) due to its efficient U-Net architecture, which is faster and more memory efficient than other diffusion generators. We accessed Imagen from https://github.com/lucidrains/imagen-pytorch. This enabled model training in approximately 28 h and inference in approximately 45 min on an NVIDIA A4000 GPU in the cross-cell-line experiment.

### 2.9 CTCF loop validation

Given a set of loops called from a Hi-C matrix, we follow the CTCF loop validation protocol used by Rao *et al.* (2014).

1. We obtained CTCF, SMC3, and RAD21 ChIP-seq experimental datasets that identify binding sites along the genome for the given transcription factor.
2. We would expect CTCF-mediated loops to co-occur with CTCF, SMC3, and RAD21 ChIP-seq peaks. For each loop spanning from locus *i* to locus *j*, we identified the *peak-associated loops*, defined as the subset of called loops that also contain a ChIP-seq peak in the loci [i,j] for all of the CTCF, SMC3, and RAD21 datasets. If *i* and *j* were fewer than 15 kb apart, we symmetrically expanded the peak search window around the anchor loci until it was 15 kb.
3. Although the presence of a CTCF ChIP-seq peak indicates an accessible CTCF binding site, it does not capture the *orientation*. We therefore leveraged DNA sequence to establish motif orientation at each of the peak-associated loops. We used FIMO (Grant *et al.* 2011) to search the human reference genome (build hg19), using a CTCF motif probability weight matrix^1^ and a *P*-value threshold of 1e−4. For each peak-associated loop, we investigated whether each 10 kb region around the anchor loci contained a CTCF motif. If a locus contained multiple nearby CTCF motifs, we assigned the motif with highest predicted likelihood from FIMO (Grant *et al.* 2011) as the loop anchor motif. Finally, if the two CTCF motifs assigned to the loop anchor points were in the convergent orientation, we marked the loop as “CTCF validated.”

For this analysis, we used the ENCODE data portal (Kagda *et al.* 2023) to access the ChIP-seq experimental datasets. For GM12878, we used four CTCF ChIP-seq datasets; for K562, we used five CTCF ChIP-seq datasets (Supplementary Section S1).

## 3 Results

### 3.1 Capricorn accurately enhances contact matrices and loop features

We first sought to evaluate Capricorn in the cross-cell-line setting, where the model is trained on the simulated low-coverage data and measured high-coverage data on one cell line and tested on the simulated low-coverage data of another cell line. We compare Capricorn to four deep learning approaches for Hi-C resolution enhancement. HiCNN (Liu and Wang 2019a) trains a deep CNN architecture with pixel-wise MSE. HiCARN-1 and HiCARN-2 (Hicks and Oluwadare 2022) both use a deep CNN architecture trained with pixel-wise MSE, total variation loss to encourage relatively smooth output images, and perceptual loss that encourages representative features computed by a separate neural network to be similar for the ground-truth target and machine-generated output; HiCARN-2 further incorporates an adversarial loss term with a GAN framework. HiCSR (Dimmick 2020) includes a pixel-wise *L*1 loss, adversarial loss, and perceptual loss. Unlike the HiCARN models, HiCSR trains a separate network directly on the target high-coverage contact matrices, rather than directly adopting a model trained on natural images.

**Figure 2.**
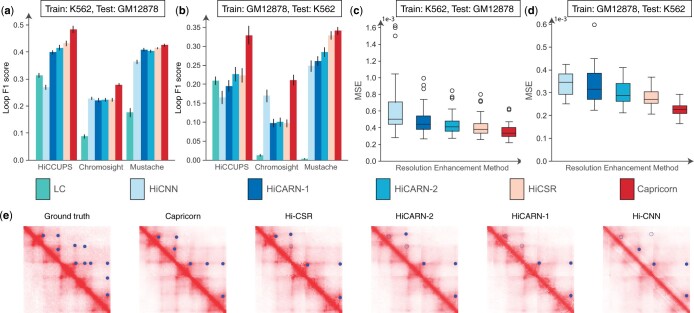
Resolution enhancement model performance. (a, b) Barplot comparison of *F*1-score for loop detection from the generated high-coverage matrices using the HiCCUPS, Chromosight, and Mustache loop calling tools (a, b, higher is better), showing the average and standard error bars by chromosome. (c, d) Boxplot comparison of the generated matrix MSE (c, d, lower is better), showing the median, interquartile range (IQR), 1.5×IQR, and outliers by chromosome. We omit the low-coverage (“LC”) baseline here as the MSE is much larger than other methods. e, Ground-truth high-coverage submatrix covering genomic loci from 47.3 Mb to 48.1 Mb on GM12878 chromosome 17, compared to the generated high-coverage submatrix output by each method. Blue circles indicated called loops in the ground-truth or predicted high-coverage data. Circles for loops that are also called in the ground truth data are filled in; loops that are called from the generated data but not the ground-truth data are empty circles

We find that Capricorn outperforms other methods both in terms of its ability to enhance chromatin loops from the low-coverage data and its accuracy in producing high-coverage data for both test cell lines in the cross-cell-line experimental setting. As shown in Fig. 2a and b, Capricorn outperforms all other approaches in terms of its ability to recognize and enhance loop chromatin structures when transferred to both the GM12878 and K562 cell lines, a pattern which holds using the HiCCUPS (Rao *et al.* 2014), Mustache (Roayaei Ardakany *et al.* 2020), or Chromosight (Matthey-Doret *et al.* 2020) loop calling tools. Capricorn has an average loop *F*1 score of 0.50 and 0.35 when tested on GM12878 and K562, respectively, with HiCCUPS, 0.28 and 0.21 for GM12878 and K562 with Chromosight, and 0.42 and 0.34 for GM12878 and K562 with Mustache. The loop *F*1 score from the Capricorn-enhanced matrix is significantly better than the next-best performing enhancement tool in five out of six settings (Bonferroni-corrected Wilcoxon signed-rank test *P*-values <1.5e−6 and <3.0e−6 for GM12878 and K562, respectively, when called with HiCCUPS; <1.5e−6 and <3.0e−6 for GM12878 and K562, respectively, when called with Chromosight; <2.2e−2 and <2.1e−1 for GM12878 and K562, respectively, when called with Mustache). These results demonstrate that Capricorn is able to successfully identify and enhance meaningful biological contacts, such as those involved in loop formation.

We observe both that GM12878 enhancement outperforms K562 enhancement, and that some loop callers lead to better performance than others, though overall performance trends largely remain similar. The performance difference for all methods between GM12878 and K562 test data can be explained by the difference in pairwise contacts in the original dataset: GM12878 has approximately five times more measured contacts than K562 (Rao *et al.* 2014). We also find that Chromosight in particular calls many more loops than the other methods, predicting nearly twice the number of loops called by HiCCUPS on high-coverage data (Supplementary Table S3), and that this difference becomes even more pronounced for computationally enhanced data (Supplementary Table S4). This may be partially attributed to the loop template’s sensitivity to slight differences between true experimental contact matrices and computationally generated contact matrices. However, our findings suggest that Capricorn’s performance relative to other resolution enhancement approaches is largely robust to the choice of loop calling method. Moving forward, we, therefore, simplify the analysis and use HiCCUPS (Rao *et al.* 2014) as the default loop detection tool.

Capricorn further achieved a lower prediction MSE than any of the comparison approaches, indicating that the additional views can also boost the overall resolution enhancement results. Figure 2c and d, shows that Capricorn has an average MSE of 3.6e−4 and 2.3e−4 for the GM12878 and K562 test cell lines respectively, relative to 4.3e−4 and 2.8e−4 for HiCSR, the best-performing comparison approach, and to 2.4e−3 and 1.1e−3 if directly using the input low-coverage data scaled by the experimental downsampling rate.

### 3.2 Small-scale chromatin features are critical to model improvement and are model-agnostic

**Figure 3.**
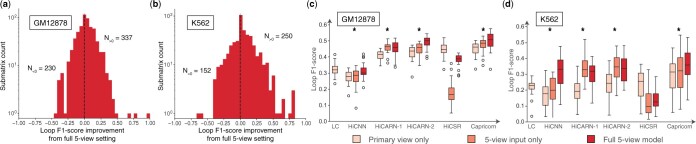
Study of Capricorn’s multiview chromatin feature framework. (a, b) Histogram of the difference in loop *F*1-score from the predicted high-coverage contact matrix in the GM12878 (a) and K562 (b) test datasets using the full five-view Capricorn framework and an alternate version of Capricorn that only includes the primary Hi-C matrix view as the input and output for the diffusion model backbone. The counts $N_{>0}$ and $N_{<0}$ indicate the number of submatrices for which the five-view setting, respectively, outperform or underperform the primary-only setting. (c, d) Boxplot comparing the performance of resolution enhancement methods in the primary-view-only setting where enhancement is only performed with Hi-C matrices, the five-view-input setting where small-scale chromatin features are included as input to the resolution model, and the full-five-view setting where small-scale chromatin features are used both as input and output to train the model backbone. “LC” indicates the results taking the low-coverage matrix and scaling all contacts by the downsampling factor 16. The boxplots show the median, IQR, 1.5× IQR, and outliers by chromosome; * indicates that the full five-view model significantly improves on the primary view model with Bonferroni-corrected one-sided paired *t*-test *P*-values <2e−3

To better attribute Capricorn’s strong performance, we next investigated the impact of including small-scale chromatin features as additional views to train the model and enhance structurally meaningful contacts. We compared Capricorn’s performance when trained to enhance chromatin features as well as the Hi-C matrix to its performance when performing resolution enhancement without any additional views. As shown in Fig. 3a and b, we find that explicitly training the model to enhance meaningful biological contacts, as captured in the small-scale chromatin features, significantly improves our ability to identify these features from the enhanced contact matrices (Bonferroni-corrected Wilcoxon signed-rank test *P*-value <2.3e−6,<4.3e−7 comparing loop *F*1-score over submatrices for test GM12878 and K562, respectively). We also test Capricorn’s performance over a subset of the input views, considering settings that incorporate one source of biological evidence (either distance-normalized contacts, loops, or TADs) in addition to the input contact matrix, and settings containing all of Capricorn’s biologically augmented views except one. We find that the full version of Capricorn performs best out of all variants in terms of the loop *F*1 score (Supplementary Table S5).

Because our multiview idea is not architecture dependent, we then tested whether the benefits of including small-scale chromatin features as part of model training generalized to other resolution enhancement network architectures. To this end, we updated each of the comparison models to accept multichannel input matrices and compared the results of three model formulations (Fig. 3c and d).

1. *Primary view*: This setting uses the comparison models’ default resolution enhancement pipelines with the low- and high-coverage Hi-C matrices as input and output. We also consider Capricorn’s performance when trained without the additional biological views in this setting.
2. *Five-view input only*: This setting uses the additional chromatin feature views as input to the model, but is still trained to only predict the high-coverage view. As shown in Fig. 3c and d, many methods perform better in this setting than the original primary view, indicating the utility of the additional biological feature inputs.
3. *Full five-view model*: This setting uses Capricorn’s complete multiview setting, including all five chromatin feature views as input and training the model to enhance the small-scale chromatin features in addition to the Hi-C matrices. This setting yields the best downstream loop calling performance for Capricorn (one-sided paired *t*-test *P*-value <0.02 relative to the five-view input-only setting) and three of the four comparison approaches. These additional performance highlight the additional value of including small-scale chromatin features as model outputs that are explicitly included in the loss function.

Although we show our key multiview idea to be generalizable to many model architectures and loss formulations, we still find that Capricorn’s diffusion model backbone outperforms the convolutional models. Comparing the full five-view model results for Capricorn and HiCARN-2 (Hicks and Oluwadare 2022), the best-performing comparison approach, Capricorn still performs better than the other enhanced approaches, with a average loop *F*1 scores of 0.50 and 0.35 for GM12878 and K562, respectively, relative to HiCARN-2’s 0.49 and 0.33 (Bonferroni-corrected one-sided paired *t*-test *P*-values <3e−3 and <5e−5, respectively). Capricorn’s multiview framework only fails to benefit downstream loop calling for HiCSR. We hypothesize that HiCSR performs poorly when including additional biological views due to its denoising autoencoder (Dimmick 2020) for perceptual loss on the generated outputs, which distinguishes its architecture from HiCARN-2’s convolutional GAN architecture.

This observation points to the broad applicability of Capricorn’s key idea, and also suggests opportunities for including additional Hi-C-derived views based on expert domain knowledge for various downstream genome folding analyses.

### 3.3 Capricorn generalizes across chromosomes

To confirm that Capricorn’s strong performance is not due to memorizing the training chromosomes with relatively small cell-line differences, we conducted a second experiment to rigorously test Capricorn in the cross-chromosome setting. Here, we withhold chromosomes 2, 6, 10, and 12 as a validation set and reserve chromosomes 4, 14, 16, and 20 as test data. We carried out two experiments to examine cross-chromosome and intra-cell-line generalization as well as cross-chromosome and cross-cell-line generalization.

**Figure 4.**
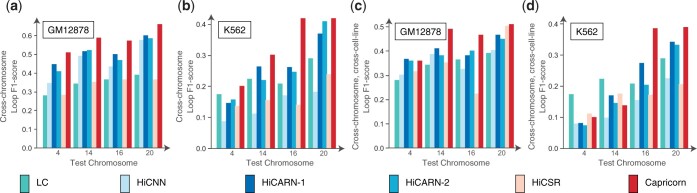
Model performance comparison in cross-chromosome and cross-chromosome, cross-cell-type experimental settings. (a–d) Barplots showing loop *F*1-score by test chromosome in a cross-chromosome experiment (a, b) and cross-chromosome, cross-cell-type experiment (c, d). “LC” indicates the results using only the low-coverage input data. Plots are labeled with the cell line of the test data

We find that Capricorn is able to transfer its learned resolution enhancement patterns to never-before-seen genomic loci in the cross-chromosome intra-cell-line setting better than other methods (Wilcoxon signed-rank test *P*-value <7.9e−3). Across the four test chromosomes, Capricorn’s generated high-coverage contact matrix had an average loop *F*1-score of 0.58 in the GM12878 experiment (Fig. 4a) and 0.35 in the K562 experiment (Fig. 4b), relative to the best-performing comparison approaches at 0.52 for HiCARN-1 and 0.26 for HiCARN-2. Similarly, Capricorn had the highest loop *F*1 scores in the cross-chromosome, cross-cell-line experiments as shown in Fig. 4c and d (Wilcoxon signed-rank test *P*-value <5.5e−2), with an average loop *F*1 score of 0.46 on GM12878 and 0.25 on K562 test data, relative to the best comparison approaches’ respective loop *F*1-scores of 0.40 and 0.21 from HiCARN-1. As in the cross-cell-line setting, all methods perform better on GM12878 test data because they contain many more measured contacts than the K562 experimental data. This result highlights Capricorn’s ability to generalize the informative, small-scale chromatin patterns it learns across genomic loci as well as cell lines, reiterating the effectiveness of diffusion-based modeling of additional chromatin feature views.

### 3.4 Loops discovered with Capricorn are enriched for convergent CTCF motifs

We further validated the loops identified from Capricorn’s generated high-coverage contact matrix with additional data not used in the resolution enhancement framework, including DNA sequence and ChIP-seq (Schmidt *et al.* 2009) experimental measurements. The CCCCTC-binding factor (CTCF) is a key protein in 3D structure determination for mammalian genomes (Handoko *et al.* 2011), and many loops anchor at CTCF motifs (Rao *et al.* 2014). In particular, cohesin-mediated loop formation is facilitated by pairs of flanking CTCF binding sites occurring in opposite orientations. As our methods do not make use of the primary DNA sequence, we can use pairs of inward-facing CTCF motifs as additional experimental evidence in support of a candidate loop.

We followed the previously described CTCF validation protocol. First, to validate that our implementation is correct, we applied the validation protocol to the high-coverage GM12878 data. We find that a similar proportion of loops are associated with convergent CTCF loops (41% in our analysis versus 42% in the original analysis). The small discrepancy in the percentages is likely due to differences in the exact ChIP-seq experimental data used, our choice of FIMO (Grant *et al.* 2011) for motif search, and the pre-processing applied to both the low- and high-coverage contact matrices in our resolution enhancement setting. However, the results are still very similar, and give us confidence in our analysis steps.

Next, we apply the CTCF validation procedure to the loops called for GM12878 and K562 across the high-coverage, low-coverage, and Capricorn-generated contact matrices. Importantly, while many chromatin loops are mediated by CTCF binding sites (Rao *et al.* 2014) non-CTCF-mediated loops also exist, so the lack of CTCF anchor motifs should not be interpreted as clear evidence of a false positive; however, we would expect the *ratio* of CTCF-mediated loops to be similar to the high-coverage data for well-enhanced data. The results (Table 2) show that the Capricorn-enhanced matrix identifies plausible loops. Notably, loops identified from Capricorn’s generated high-coverage matrices have very similar rates of CTCF support to the loops called from the experimental high-coverage data for both GM12878 and K562, with 41.1% support for high-coverage-based loops compared to 42.8% support for Capricorn-based-loops in GM12878 and 37.6% support for high-coverage-based loops compared to 36.8% support for Capricorn-based loops in K562. By comparison, loops called from the low-coverage data exhibit much lower levels of CTCF support, suggesting that the loop caller’s false discovery rate is not well controlled with such sparse contact matrices. This finding is especially important, because it indicates that loop calling results produced from low-coverage data without a resolution enhancement tool may be problematic. Hence, our results further support the need for resolution enhancement methods like Capricorn, which can produce denser contact matrices that are compatible with existing loop calling algorithms.

A further analysis of the CTCF support for loops called by Mustache (Roayaei Ardakany *et al.* 2020) and Chromosight (Matthey-Doret *et al.* 2020) as well as CTCF validation following the procedure in Roayaei Ardakany *et al.* (2020) is provided in Supplementary Section S9.

## 4 Discussion

In this work, we present Capricorn as a tool for Hi-C resolution enhancement. Capricorn explicitly models the biology underlying experimental contact matrices by incorporating small-scale chromatin features into the model formulation and loss function. Furthermore, we find that this key insight is widely applicable, improving performance for three out of four comparison approaches. However, the small-scale chromatin feature views still perform best with Capricorn’s conditional diffusion model backbone.

We demonstrate Capricorn’s strong performance in cross-cell-line, cross-chromosome, and cross-chromosome-cross-cell-line settings with the GM12878 and K562 datasets. In all three measured settings, Capricorn is best able to generate high-coverage, high-resolution data containing accurate chromatin loops that can be called with standard tools (Rao *et al.* 2014, Matthey-Doret *et al.* 2020, Roayaei Ardakany *et al.* 2020). This highlights Capricorn’s generalizability as well as the value of including additional biological data views, differentiating Hi-C resolution enhancement from super-resolution in natural image applications. Finally, we leverage DNA sequence to further validate the loops identified from Capricorn’s generated data and find CTCF support for Capricorn-based loops similar to the experimentally generated high-coverage-based loops.

In the future, Capricorn’s framework can be further broadened to include additional biological views designed specifically for downstream tasks of interest. In particular, new views that contain structural information covering a more than 400^2^ kb locus could help enhancement for TADs and A/B compartments. Such analyses could also test the relevance of various biological views on different tasks. We also imagine that future work will study the impact of even more model backbones on the multiview resolution enhancement problem.

Future work can further study Capricorn’s generalizability. Here, we have focused on two human cell lines measured with *in situ* Hi-C. Follow-up work can apply Capricorn to more Hi-C cell line data and study the impact of training data size. This work could also apply Capricorn to a cross-species transfer learning setting, such as applying the model to mouse data after training only on human data. Another generalizability study could apply a model trained on *in situ* Hi-C data to other experimental contact matrix types, such as micro-C (Hsieh *et al.* 2015, 2020).

## Supplementary Material

btae211_Supplementary_Data

## Data Availability

The data underlying this article are available from the Gene Expression Omnibus (GEO) with accession codes GSE63525 and GSM1872886, and from the ENCODE data portal with accession codes ENCFF833FTF, ENCFF002DAJ, ENCFF473RXY, ENCFF710VEH, ENCFF002CPK, ENCFF753RGL, ENCFF686FLD, ENCFF002DDJ, ENCFF738TKN, ENCFF002CEL, ENCFF002DBD, ENCFF085HTY, ENCFF002CXU, and ENCFF041YQC.

## Footnotes

1
https://jaspar.elixir.no/api/v1/matrix/MA0139.1.meme.
