# Supplementary material for: Enhancing Hi-C contact matrices for loop detection with Capricorn: a multiview diffusion model
Source: Bioinformatics. 2024 Jun 28;40(Suppl 1):i471–80. doi: 10.1093/bioinformatics/btae211 (PMC11211821; doi:10.1093/bioinformatics/btae211)
Supplement: btae211_Supplementary_Data [file btae211_supplementary_data.pdf]

Supplemental Information

Enhancing Hi-C contact matrices for loop detection with Capricorn, a multi-view diffusion model

T. Fang, Y. Liu, A. Woicik, M. Lu, A. Jha, X. Wang, G. Li, B. Hristov, Z. Liu, H. Xu, W. Noble, S. Wang

March 18, 2024

Contents

S1 Dataset accessibility **2**
- S1.1 Hi-C experimental data . . . 2
- S1.2 CTCF validation ChIP-seq data . . . 2

S2 Additional biological views in Capricorn **2**
- S2.1 Distance-corrected view . . . 2
- S2.2 Chromatin loops view . . . 3
- S2.3 TAD score view . . . 3

S3 Multi-view weighting details **3**

S4 Diffusion models **4**
- S4.1 Conditional diffusion models . . . 4

S5 Cross-cell-line loop statistics **4**

S6 Cross-chromosome loop statistics **5**

S7 Cross-cell-line, cross-chromosome loop statistics **6**

S8 Capricorn view ablation study **6**

S9 Additional CTCF validation **6**
- S9.1 CTCF validation for loops called with Mustache . . . 7
- S9.2 CTCF validation for loops called with Chromosight . . . 7
- S9.3 Secondary CTCF validation . . . 7

Cell line	Transcription factor	ENCODE accession code
GM12878	CTCF	ENCFF833FTF<br>ENCFF002DAJ<br>ENCFF473RXY<br>ENCFF710VEH
	RAD21	ENCFF002CPK<br>ENCFF753RGL
	SMC3	ENCFF686FLD
K562	CTCF	ENCFF002DDJ<br>ENCFF738TKN<br>ENCFF002CEL<br>ENCFF002DBD<br>ENCFF085HTY
	RAD21	ENCFF002CXU
	SMC3	ENCFF041YQC

Table S1: Accession codes for the ChIP-seq data used in the CTCF loop validation analysis following the protocol described in Rao et al. [2].

S1 Dataset accessibility

S1.1 Hi-C experimental data

We collect the GM12878 and K562 cell line Hi-C experimental data from the Gene Expression Omnibus (GEO) database [1] with accession code GSE63525.

S1.2 CTCF validation ChIP-seq data

For the CTCF loop validation following Rao et al. [2] we use the ENCODE data portal [3] to access several ChIP-seq datasets. We provide the accession codes for these experiments in Table S1. In our secondary CTCF validation conducted following the protocol in Roayaei Ardakany et al. [4] and provided in Supplementary Section S9.3, we use the GEO accession code GSM1872886 to obtain CTCF ChIP-seq data.

S2 Additional biological views in Capricorn

Here, we provide more precise definitions of how Capricorn's additional biological views are computed.

S2.1 Distance-corrected view

Capricorn includes a view that accounts for the fact that nearby loci are more likely to interact by chance than more distal loci, such as the strong number of contacts observed along the diagonal of an uncorrected Hi-C matrix. Therefore, one common pre-processing step for Hi-C analyses involves correcting for this proximity bias [5–7].

Consider the input, low-coverage Hi-C contact matrix $\mathbf{X} \in \mathbb{N}^{l \times l}$, where $l = \lfloor \frac{L}{\Delta} \rfloor$ indicates the number of binned genomic loci in the matrix. The distance-correction module $D : \mathbf{X} \to \mathbf{X}^{(oe)}$ is defined as follows. First, compute a distance-based expected matrix by taking the contacts for all pairs of loci (i, j) that are $|i - j|$ apart in the genome. In practice, this is computed by taking the average along each diagonal of the contact matrix as

$$\mathcal{E}(\mathbf{X})[i, j] = \frac{1}{l - |i - j|} \sum_{k=0}^{l-|i-j|} \mathbf{X}[k, k + |i - j|] \tag{S1}$$

This can be repeated for all $i \in [1, \dots, n]$ and $j \in [1, \dots, n]$ to produce $\mathcal{E}(\mathbf{X}) \in \mathbb{R}^{l \times l}$. After observing that most distance-expected values occurred in the range $[0, 16]$, we further clamp and normalize a distance-corrected version of the input matrix as

$$\mathbf{X}^{(oe)}[i, j] = \begin{cases} 0 & \text{if } \mathcal{E}(\mathbf{X})[i, j] = 0 \\ \frac{\min\left(\frac{\mathbf{X}[i,j]}{\mathcal{E}(\mathbf{X})[i,j]}, 16\right)}{16} & \text{otherwise.} \end{cases} \tag{S2}$$

We follow the same steps to compute $\mathcal{E}(\mathbf{Y})$ and $\mathbf{Y}^{(oe)}$ given the high-coverage contact matrix $\mathbf{Y}$.

S2.2 Chromatin loops view

We include two additional views based on chromatin loop structure identified from the input contact matrices. We use the Hi-C Computational Unbiased Peak Search (HiCCUPS) loop calling tool [2]. HiCCUPS tests whether the measured contacts for possible (i, j) loop anchor points are significantly more enriched than the surrounding contacts.

Specifically, the method combines a 10×10 donut kernel centered at a specific locus with horizontal, vertical, and lower-left quadrant kernels as described in Rao et al. [2]. For a given locus (i, j), we use the distance-based expected matrix as defined in (S1) to compute

$$\alpha_p = \kappa_p * \mathbf{X} \qquad \beta_p = \kappa_p * \mathcal{E}(\mathbf{X}) \tag{S3}$$

for each kernel $\kappa_p = \{\kappa_1, \ldots, \kappa_4\}$. Then we compute the *loop ratio* for the input matrix as

$$\mathbf{X}^{(loop-r)} = \min_{p \in \{1,\ldots,4\}} \frac{\beta_p \mathbf{X}}{\alpha_p \mathcal{E}(\mathbf{X})}. \tag{S4}$$

We follow the HiCCUPS algorithm and fit a Poisson distribution to the observed measurements with λ-chunking, where we set $\lambda = 2^{1/3}$ as in the default HiCCUPS settings [2]. In the λ-chunking protocol, we define $\mu_{i,j} = \max_c \lambda^c$ subject to $\frac{\alpha_p}{\beta_p}\mathcal{E}(\mathbf{X})[i,j] < \lambda^c$, grouping pixels into relative intensity ranges of $\{[0, 1), [1, 2^{1/3}), [2^{1/3}, 2^{2/3}), \ldots\}$. Finally, we use the result of λ-chunking to compute the *loop p-value* as

$$\mathbf{X}^{(loop-p)}[i,j] = \max_{p \in \{1,\ldots,4\}} \mathtt{BH}_{\mu_{i,j}} \left(f(\mathbf{X}[i,j] \geq t \middle| \mu = \mu_{i,j}) \right), \tag{S5}$$

where $f(\cdot \geq t|\mu)$ represents the survival function of the Poisson distribution with expected value μ, and $\mathtt{BH}_{\mu_{i,j}}$ indicates the Benjamini-Hochberg false discovery rate correction [8, 9] over the p-values for all pixels with the same λ-chunked $\mu_{i,j}$. We compute $\mathbf{Y}^{(loop-r)}$ and $\mathbf{Y}^{(loop-p)}$ based on the ground-truth high-coverage matrix $\mathbf{Y}$ in an analogous manner.

S2.3 TAD score view

We compute the insulation score (IS) [10] for TAD detection, using sliding windows along the contact matrix diagonal to identify insulated regions with low scores and in-domain regions with high scores. We then smooth the IS scores by averaging them over a 21-bin domain and assign the within-TAD label 1 to contiguous regions with monotonically decreasing ISs centered around some locus (i, j), where all ISs are still larger than the contact matrix average IS and the IS of (i, j) is at least 10% larger than the IS at the region boundary.

S3 Multi-view weighting details

Given the input, multi-view low-coverage contact matrix representation $\tilde{\mathbf{X}}(\mathbf{X}) \in \mathbb{R}^{5 \times L/\Delta \times L/\Delta}$, we computed an initial view weight vector $\omega_0 \in \mathbb{R}+^5$ as

$$\omega_0[v] = \sqrt{\frac{1}{|\Xi|} \sum_{\xi \in \Xi} \frac{\mathtt{var}(\tilde{\mathbf{X}}(\mathbf{X}_\xi)[0]}{\mathtt{var}(\tilde{\mathbf{X}}(\mathbf{X}_\xi)[v]}} \tag{S6}$$

for the vth view, where $\mathtt{var}(\cdot)$ denotes variance, Ξ indicates the set of all chromosomes tested for the given cell line, and $\tilde{\mathbf{X}}(\mathbf{X}_\xi)$ indicates the views computed from the low-coverage cis-contact matrix $\mathbf{X}$ for chromosome ξ. The computed ω_0 for both GM12878 and K562 is reported in Table S2.

We then further refine the weights to account for the difficulty of generating the high-resolution version of each view. Specifically, we compute a view-specific difficulty score using the validation set, as

$$d[v] = \mathtt{mse}\left(f\left(\sqrt{\omega_0}\tilde{\mathbf{X}}(\mathbf{X}^{\mathbf{val}})\right)[v], (\omega_0 \tilde{\mathbf{Y}}(\mathbf{Y}^{\mathbf{val}})[v])\right), \tag{S7}$$

where $\mathtt{mse}(\cdot, \cdot)$ indicates the mean squared error, $f(\cdot)$ is the diffusion model backbone, and $(\mathbf{X}^{\mathbf{val}}, \mathbf{Y}^{\mathbf{val}})$ are the paired low- and high-resolution contact matrices in the validation set. Finally, we compute $\omega \in \mathbb{R}+^5$ as

$$\omega[v] = \sqrt{\frac{\min_{u \in \{0,\ldots,4\}]} d[u]}{d[v]}} \text{ for } v = 1, \ldots, 4 \qquad \omega[0] = 1. \tag{S8}$$

We explicitly set the weight of the 0th view, which contains the low-coverage input contact matrix without further alteration, to 1. This avoids altering the basic matrix view that we aim to enhance. The precise weight vector ω is given in Table S2 for both GM12878-based training and K562-based training.

During all subsequent training runs, we more precisely compute the input as

$$\tilde{\mathbf{X}}(\mathbf{X}|\omega) = \left[\sqrt{\omega[0]}\mathbf{X}, \sqrt{\omega[1]}\mathbf{X}^{(oe)}, \sqrt{\omega[2]}\mathbf{X}^{(tad)}, \sqrt{\omega[3]}\mathbf{X}^{(loop-p)}, \sqrt{\omega[4]}\mathbf{X}^{(loop-r)}\right] \tag{S9}$$

$$\tilde{\mathbf{Y}}(\mathbf{Y}|\omega) = \left[\sqrt{\omega[0]}\mathbf{Y}, \sqrt{\omega[1]}\mathbf{Y}^{(oe)}, \sqrt{\omega[2]}\mathbf{Y}^{(tad)}, \sqrt{\omega[3]}\mathbf{Y}^{(loop-p)}, \sqrt{\omega[4]}\mathbf{Y}^{(loop-r)}\right]. \tag{S10}$$

For simplicity of notation, we refer to this as $\tilde{\mathbf{X}}(\mathbf{X})$ and $\tilde{\mathbf{Y}}(\mathbf{Y})$ in the manuscript for all model training after the initial weight tuning.

S4 Diffusion models

Diffusion models leverage a latent Markov chain framework that iteratively denoises an input to produce diverse and realistic outputs [11, 12]. The models are split into a T-step *forward process* $q(\mathbf{y}_t|\mathbf{y}_{t-1})$ and T-step *reverse process* $p(\mathbf{y}_{t-1}|\mathbf{y}_t, \mathbf{x})$, where $\mathbf{y}_0 \in \mathbb{R}^{C\times W\times H}$ is the C-channel original, high-fidelity image, $\mathbf{y}_t \in \mathbb{R}^{C\times W\times H}$ is a latent variable, and $\mathbf{x}$ is an optional input term on which to condition the process. Specifically, the forward process computes progressively noisier latent representations of the true image as

$$q(\mathbf{y}_t|\mathbf{y}_{t-1}) \sim \mathcal{N}(\sqrt{\alpha_t}\mathbf{y}_{t-1}, \beta_t\mathbf{I}) \tag{S11}$$

for $t = 1, \ldots, T$, where β_t is defined by a noise schedule, such as a cosine schedule [13, 14], and $\alpha_t = 1 - \beta_t$. The reverse diffusion process, optionally conditioned on input $\mathbf{x}$, can be then parameterized by a learnable $\epsilon_\theta(\cdot)$ as

$$p_\theta(\mathbf{y}_{t-1}|\mathbf{y}_t, \mathbf{x}, t) = \mathcal{N}\left(\frac{1}{\sqrt{\alpha_t}}\left(\mathbf{y}_t - \frac{\beta_t}{\sqrt{1-\prod_{s=1}^{t}\alpha_s\epsilon_\theta(\mathbf{y}_t, \mathbf{x}, t)}}\right), \beta_t\mathbf{I}\right), \tag{S12}$$

where $\epsilon_\theta(\cdot)$ is trained to predict noise from the previous step's $\mathbf{y}_t$ prediction and the conditional input $\mathbf{x}$. We denote a sample from a step of the reverse process as $\hat{\mathbf{y}}_{t-1} \sim p_\theta(\mathbf{y}_{t-1}|\mathbf{y}_t, \mathbf{x}, t)$, or $\hat{\mathbf{y}}_{t-1} \sim p_\theta$ for brevity of notation.

S4.1 Conditional diffusion models

We focus on conditional diffusion models that are trained with a MSE objective in the image's pixel space, or the bin space of a contact matrix. In this case, we compute the loss

$$\mathcal{L}(\theta) = \mathbb{E}_{(\mathbf{x},\mathbf{y}_0)}\left[\mathbb{E}_{t\in\{1,\ldots,T\}}\left[w_t\mathbb{E}_{\mathbf{y}_t|\mathbf{y}_0}\left[\mathbb{E}_{\hat{\mathbf{y}}_{t-1}\sim p_\theta}\left[\|\hat{\mathbf{y}}_{t-1} - \mathbf{y}_{t-1}\|_2^2\right]\right]\right]\right], \tag{S13}$$

where w_t is a diffusion loss hyperparameter impacting the the relative amount of sampled Gaussian noise [14]. The expectation over $t \in \{1, \ldots, T\}$ reflects how we can compute loss at a different number of steps of the reverse diffusion process, introducing t noise samples to the target $\mathbf{y}_0$ during the forward process before denoising.

S5 Cross-cell-line loop statistics

In addition to the loop F1 score, here we report the total number of loops called and the precision and recall of the called loops, still treating loops called from the high-coverage data as the ground truth. We report results using the HiCCUPS [2], Mustache [4], and Chromosight [15] loop calling tools for evaluation; in all cases, Capricorn's loop-based views were computed using the HiCCUPS algorithm. For reference, the total number of loops called from the original high-coverage Hi-C matrices is provided in Table S3. The performance metrics for all three loop calling algorithms given the low-coverage (LC) data and the enhanced matrices produced by Capricorn (ours), HiCNN [16], HiCARN-1 [17], HiCARN-2 [17], and HiCSR [18] are shown in Table S4.

Cell line	Weight vector	$\mathbf{X}$ $(v=0)$	$\mathbf{X}^{(oe)}$ $(v=1)$	$\mathbf{X}^{(tad)}$ $(v=2)$	$\mathbf{X}^{(loop-p)}$ $(v=3)$	$\mathbf{X}^{(loop-r)}$ $(v=4)$
GM12878	ω_0	1.00	7.30e−2	9.83e−3	1.59e−1	3.10e−2
	ω	1.00	7.30e−2	8.44e−3	3.81e−2	1.82e−2
K562	ω_0	1.00	3.58e−2	9.99e−3	1.37e−1	2.29e−2
	ω	1.00	3.58e−2	9.69e−3	4.92e−2	1.39e−2

Table S2: Capricorn's computed weights for each view when training on either GM12878 or K562. The initial, distribution-based weights ω_0 and further refined, difficulty-based weights ω are reported. For training the final model, we use the ω weight vector.

Cell line	Loop caller	Loops called
GM12878	HiCCUPS	10,176
	Mustache	15,417
	Chromosight	22,648
K562	HiCCUPS	5136
	Mustache	7776
	Chromosight	10,630

Table S3: Number of loops called from the original high-coverage data across loop calling methods and cell lines.

Cell line	Loop caller	Method	Loops called	F1 score	Precision	Recall
GM12878	HiCCUPS	LC	7021	0.32	0.41	0.27
		HiCNN	10,625	0.28	0.28	0.28
		HiCARN-1	9035	0.41	0.44	0.39
		HiCARN-2	8793	0.43	0.46	**0.40**
		HiCSR	7608	0.45	0.53	0.40
		Capricorn	5686	**0.50**	**0.70**	0.39
	Mustache	LC	1483	0.18	**0.97**	0.10
		HiCNN	37,218	0.36	0.25	0.64
		HiCARN-1	34,236	0.40	0.29	0.66
		HiCARN-2	36,301	0.40	0.28	**0.68**
		HiCSR	34,782	0.41	0.29	**0.68**
		Capricorn	33,109	**0.42**	0.30	**0.68**
	Chromosight	LC	1495	0.09	**0.68**	0.05
		HiCNN	87,693	0.23	0.15	0.53
		HiCARN-1	100,370	0.22	0.14	0.56
		HiCARN-2	101,953	0.23	0.14	0.57
		HiCSR	100,011	0.22	0.14	0.57
		Capricorn	83,058	**0.28**	0.18	**0.61**
K562	HiCCUPS	LC	4668	0.22	0.28	0.20
		HiCNN	9117	0.17	0.20	0.20
		HiCARN-1	7879	0.20	0.19	0.24
		HiCARN-2	5406	0.24	0.27	0.23
		HiCSR	4675	0.23	0.31	0.21
		Capricorn	2144	**0.35**	**0.60**	**0.25**
	Mustache	LC	17	0.00	**0.36**	0.00
		HiCNN	30,870	0.24	0.15	0.63
		HiCARN-1	28,283	0.25	0.16	**0.63**
		HiCARN-2	25,099	0.28	0.18	0.62
		HiCSR	19,656	0.32	0.22	0.60
		Capricorn	18,080	**0.34**	0.24	0.59
	Chromosight	LC	267	0.01	**0.24**	0.01
		HiCNN	48,557	0.17	0.11	0.43
		HiCARN-1	101,468	0.10	0.06	0.45
		HiCARN-2	95,614	0.10	0.06	0.44
		HiCSR	105,196	0.10	0.06	0.46
		Capricorn	43,390	**0.21**	0.14	**0.52**

Table S4: Cross-cell-line resolution enhancement performance based on identifiable loops using different loop calling methods and test cell lines. "LC" indicates the low-coverage contact map provided as input to the resolution enhancement methods. The best-performing resolution enhancement method for each cell line and loop caller is shown in bold for each metric. Reported performance numbers are the average performance across test chromosomes (23 chromosomes for GM12878; 22 chromosomes for K562).

S6 Cross-chromosome loop statistics

In addition to the loop F1 score, here we report the total number of loops called and the precision and recall of the called loops in the cross-chromosome experimental setting, still treating loops called from the high-coverage data as the ground truth. We report results in Table S5 using the HiCCUPS [2] loop calling tools for evaluation.

Cell line	Method	Loops called	F1 score	Precision	Recall
GM12878	LC	1086	0.35	0.43	0.29
	HiCNN	803	0.46	0.65	0.37
	HiCARN-1	845	0.52	0.69	0.42
	HiCARN-2	945	0.50	0.63	0.42
	HiCSR	552	0.34	0.61	0.24
	Capricorn	754	**0.58**	**0.83**	**0.46**
K562	LC	548	0.22	0.29	0.19
	HiCNN	453	0.14	0.19	0.11
	HiCARN-1	483	0.26	0.35	0.22
	HiCARN-2	587	0.26	0.31	0.23
	HiCSR	819	0.17	0.18	0.17
	Capricorn	264	**0.35**	**0.64**	**0.25**

Table S5: Cross-chromosome resolution enhancement based on identifiable loops using the HiCCUPS [2] loop caller. "LC" indicates the low-coverage contact map provided as input to the resolution enhancement methods. The best-performing resolution enhancement method for each cell line and each metric is bolded. Reported performance numbers are the average performance across 4 test chromosomes.

Test cell line	Method	Loops called	F1 score	Precision	Recall
GM12878	HiCNN	1040	0.14	0.18	0.15
	HiCARN-1	1006	0.22	0.24	0.23
	HiCARN-2	1520	0.19	0.21	**0.24**
	HiCSR	204	0.17	0.39	0.11
	Capricorn	289	**0.25**	**0.44**	0.18
K562	HiCNN	2150	0.36	0.31	0.45
	HiCARN-1	1210	0.41	0.45	**0.39**
	HiCARN-2	1316	0.40	0.42	**0.39**
	HiCSR	1665	0.35	0.36	0.37
	Capricorn	864	**0.46**	**0.60**	0.37

Table S6: Cross-cell-line, cross-chromosome resolution enhancement based on identifiable loops using the HiCCUPS [2] loop caller. The best-performing resolution enhancement method for each cell line and each metric is bolded. Reported performance numbers are the average performance across 4 test chromosomes.

S7 Cross-cell-line, cross-chromosome loop statistics

In addition to the loop F1 score, here we report the total number of loops called and the precision and recall of the called loops in the cross-cell-line, cross-chromosome experimental setting, still treating loops called from the high-coverage data as the ground truth. We report results in Table S6 using the HiCCUPS [2] loop calling tools for evaluation. Note that there is no comparison with the low-coverage input baseline in this setting, as the low-coverage for the test cell line is not available to the enhancement models during inference.

S8 Capricorn view ablation study

In addition to applying our multi-view framework to the other comparison approaches, we also conducted an ablation study over the views included in Capricorn. Specifically, we considered the 8 different approaches listed in Table S7, still using Capricorn's diffusion model backbone for resolution enhancement.

We then compared the loop F1 score and MSE in the cross-cell-line setting for all ablated methods. Results are given in Table S8.

S9 Additional CTCF validation

We repeat the CTCF validation procedure described in Rao et al. [2] for loops called with other loop calling methods. As in the CTCF validation analysis with HiCCUPS loop calls, we compare the percent of loops with flanking convergent CTCF motifs for high-coverage, low-coverage, and Capricorn-enhanced contact matrices.

In general, we would expect that well-enhanced contact matrices produce loop calls with similar levels of CTCF support to the high-coverage experimental data. However, manual inspection of Mustache [4] and Chromosight

Ablation study name	$\mathbf{X}$	$\mathbf{X}^{(oe)}$	$(\mathbf{X}^{(loop-p)}, \mathbf{X}^{(loop-r)})$	$\mathbf{X}^{(tad)}$
HiC	✓			
HiC+Distance	✓	✓		
HiC+Loop	✓		✓	
HiC+TAD	✓			✓
Full−Distance	✓		✓	✓
Full−Loop	✓	✓		✓
Full−TAD	✓	✓		✓
Full	✓	✓	✓	✓

Table S7: Ablation studies conducted over Capricorn's multi-view framework. We considered combining the original Hi-C contact matrices with only one additional source of information, as well the the full Capricorn framework missing only one source of information.

Cell line	Study name	Loop F1 score
GM12878	HiC	0.451*
	HiC+Distance	0.468*
	HiC+Loop	0.494
	HiC+TAD	0.462*
	Full−Distance	0.498
	Full−Loop	0.479*
	Full−TAD	0.474*
	Full	**0.500**
K562	HiC	0.289*
	HiC+Distance	0.325*
	HiC+Loop	0.334*
	HiC+TAD	0.340
	Full−Distance	0.337*
	Full−Loop	0.312*
	Full−TAD	0.351
	Full	**0.352**

Table S8: Comparison of different view ablation study results in the Capricorn framework for both the GM12878 and K562 cell lines. Experiments are conducted in the cross-cell-line setting. The average chromosome loop F1 score using the HiCCUPS loop calling algorithm is shown. $*$ indicates that the result is significantly worse than the complete Capricorn framework (full; Wilcoxon signed-rank test with Benjamini-Hochberg [8] correction with a controlled FDR of 0.1). The best-performing Capricorn variant is shown in bold.

[15] loops suggests that both of these loop calling methods struggle more with enhanced matrices in sparse regions, identifying loop peaks where none appear to exist (Fig. S1).

S9.1 CTCF validation for loops called with Mustache

When comparing CTCF support of loops called by Mustache [4], we report both the overall support for all loops identified in the Capricorn-enhanced contact matrices and the support for the k highest-confidence loop calls, where k is selected as the number of loops called from the high-coverage data and confidence is determined by FDR.

S9.2 CTCF validation for loops called with Chromosight

When comparing CTCF support of loops called by Chromosight [15], we report both the overall support for all loops identified in the Capricorn-enhanced contact matrices and the support for the k highest-confidence loop calls, where k is selected as the number of loops called from the high-coverage data and confidence is determined by Chromosight's loop score.

S9.3 Secondary CTCF validation

In addition to the CTCF-motif-based loop validation following the protocol of Rao et al. [2], here we also provide a CTCF-based validation following the steps in Roayaei Ardakany et al. [4] for GM12878. We downloaded a

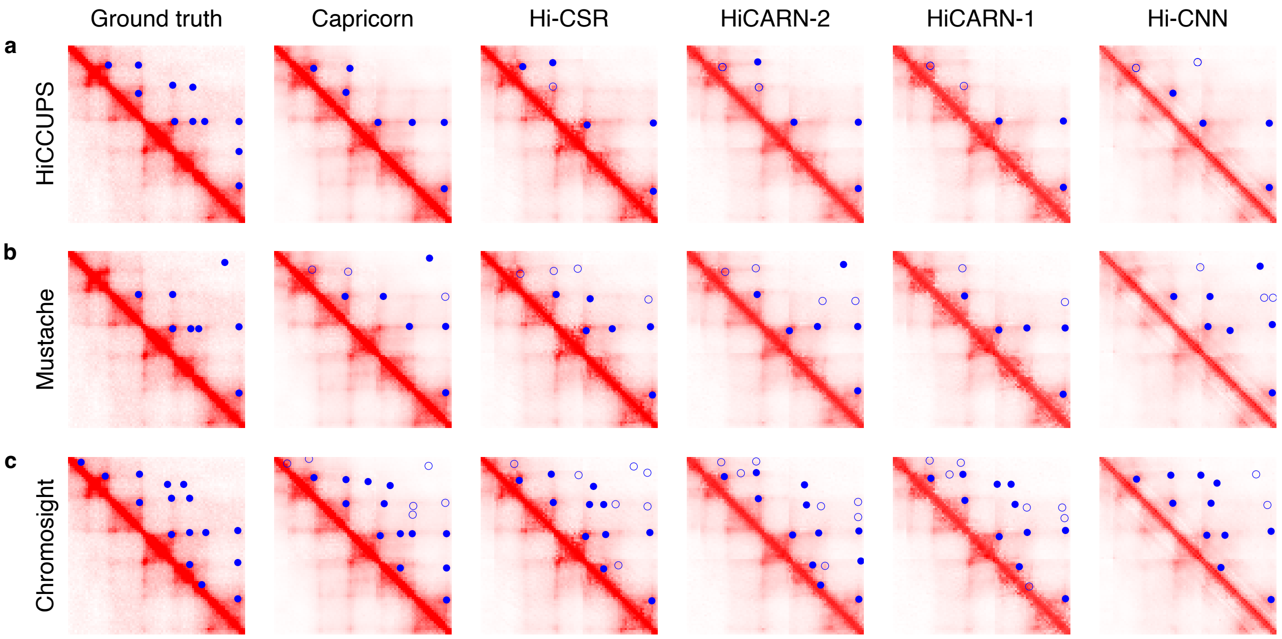

Figure S1: **Sample loop calls across resolution enhancement methods and loop callers. a-c**, Comparison of ground-truth and computationally-enhanced submatrices covering genomic loci from 47.3 Mb to 48.1 Mb on GM12878 chromosome 17. Blue circles indicated called loops in the ground-truth or predicted high-coverage data using the HiCCUPS (**a**), Mustache (**b**), and Chromosight (**c**) loop calling tools. Circles for loops that are also called in the ground truth data are filled in; loops that are called from the generated data but not the ground-truth data are empty circles.

Cell line	Contact matrix	Total loops	CTCF-validated loops	Validation rate
GM12878	High-coverage	14,430	4044	28.0%
	Capricorn (all loops)	33,109	3417	10.3%
	Capricorn (top-k)	14,430	2676	18.5%
	Low-coverage	1440	719	49.9%
K562	High-coverage	6956	2004	28.8%
	Capricorn (all loops)	18,080	1428	7.9%
	Capricorn (top-k)	6956	1091	15.7%
	Low-coverage	29	17	58.6%

Table S9: **Analysis of CTCF validation for loops called with Mustache.** We show the total number of called loops, loops also supported by flanking convergent CTCF motifs in the DNA sequence, and percentage of loops that were validated. Top-k indicates that the analysis was restricted to the k loops that Mustache called most confidently.

Cell line	Contact matrix	Total loops	CTCF-validated loops	Validation rate
GM12878	High-coverage	23,362	5635	24.1%
	Capricorn (all loops)	83,058	5126	6.2%
	Capricorn (top-k)	23,362	3657	15.7%
	Low-coverage	1504	516	34.3%
K562	High-coverage	10,887	2757	25.3%
	Capricorn (all loops)	43,390	2203	5.1%
	Capricorn (top-k)	10,887	1627	14.9%
	Low-coverage	288	41	14.2%

Table S10: **Analysis of CTCF validation for loops called with Chromosight.** We show the total number of called loops, loops also supported by flanking convergent CTCF motifs in the DNA sequence, and percentage of loops that were validated. Top-k indicates that the analysis was restricted to the k loops that Chromosight called most confidently.

GM12878 CTCF ChIA-PET [19] experiment[1] from GEO. The ChIA-PET experimental data identified 92,807 total peaks, indicating CTCF binding sites along the genome.

In this analysis, we considered a called loop to be "CTCF validated" if there was a CTCF ChIA-PET peak within 10 kb of the loop locus. Loops from the high-coverage, low-coverage, and Capricorn-enhanced contact matrices were called with HiCCUPS [2]. Results are shown in Table S11.

Method	Loops called	CTCF-validated loops
High-coverage	10,176	8370
Low-coverage	7021	2665
Capricorn	5686	3536

Table S11: Loop CTCF validation with CTCF ChIA-PET experimental validation, following the protocol in Roayaei Ardakany et al. [4].

[1]GEO accession code: GSM1872886.

References

[1] Tanya Barrett et al. NCBI GEO: archive for functional genomics data sets–update. *Nucleic Acids Res.*, 41 (Database issue):D991–5, January 2013.

[2] Suhas S P Rao et al. A 3D map of the human genome at kilobase resolution reveals principles of chromatin looping. *Cell*, 159(7):1665–1680, December 2014.

[3] Meenakshi S Kagda et al. Data navigation on the ENCODE portal. April 2023.

[4] Abbas Roayaei Ardakany et al. Mustache: multi-scale detection of chromatin loops from Hi-C and Micro-C maps using scale-space representation. *Genome Biol.*, 21(1):256, September 2020.

[5] Geoff Fudenberg et al. Predicting 3D genome folding from DNA sequence with akita. *Nat. Methods*, 17(11): 1111–1117, November 2020.

[6] Rui Yang et al. Epiphany: predicting Hi-C contact maps from 1D epigenomic signals. *Genome Biol.*, 24(1): 134, June 2023.

[7] Jian Zhou. Sequence-based modeling of three-dimensional genome architecture from kilobase to chromosome scale. *Nat. Genet.*, 54(5):725–734, May 2022.

[8] Yoav Benjamini and Yosef Hochberg. Controlling the false discovery rate: A practical and powerful approach to multiple testing. *J. R. Stat. Soc. Series B Stat. Methodol.*, 57(1):289–300, 1995.

[9] T Tony Cai and Wenguang Sun. Simultaneous testing of grouped hypotheses: Finding needles in multiple haystacks. *J. Am. Stat. Assoc.*, 104(488):1467–1481, December 2009.

[10] Emily Crane et al. Condensin-driven remodelling of X chromosome topology during dosage compensation. *Nature*, 523(7559):240–244, July 2015.

[11] Jonathan Ho et al. Denoising diffusion probabilistic models. In H Larochelle et al., editors, *Advances in Neural Information Processing Systems*, volume 33, pages 6840–6851. Curran Associates, Inc., 2020.

[12] Jascha Sohl-Dickstein et al. Deep unsupervised learning using nonequilibrium thermodynamics. In Francis Bach and David Blei, editors, *Proceedings of the 32nd International Conference on Machine Learning*, volume 37 of *Proceedings of Machine Learning Research*, pages 2256–2265, Lille, France, 2015. PMLR.

[13] Alex Nichol and Prafulla Dhariwal. Improved denoising diffusion probabilistic models. February 2021.

[14] Chitwan Saharia et al. Photorealistic Text-to-Image diffusion models with deep language understanding. In Alice H Oh, Alekh Agarwal, Danielle Belgrave, and Kyunghyun Cho, editors, *Advances in Neural Information Processing Systems*, 2022.

[15] Cyril Matthey-Doret et al. Computer vision for pattern detection in chromosome contact maps. *Nat. Commun.*, 11(1):5795, November 2020.

[16] Tong Liu and Zheng Wang. HiCNN: a very deep convolutional neural network to better enhance the resolution of Hi-C data. *Bioinformatics*, 35(21):4222–4228, November 2019.

[17] Parker Hicks and Oluwatosin Oluwadare. HiCARN: resolution enhancement of Hi-C data using cascading residual networks. *Bioinformatics*, 38(9):2414–2421, April 2022.

[18] Michael Dimmick. *HiCsr: A Hi-C Super-Resolution Framework for Producing Highly Realistic Contact Maps.* PhD thesis, University of Toronto (Canada), 2020.

[19] Guoliang Li et al. Chromatin interaction analysis with Paired-End tag (ChIA-PET) sequencing technology and application. *BMC Genomics*, 15 Suppl 12(Suppl 12):S11, December 2014.
